# Impact of Tablet Size and Shape on the Swallowability in Older Adults

**DOI:** 10.3390/pharmaceutics15041042

**Published:** 2023-03-23

**Authors:** Henriette Hummler, Cordula Stillhart, Lisa Meilicke, Michael Grimm, Elischa Krause, Marwan Mannaa, Maik Gollasch, Werner Weitschies, Susanne Page

**Affiliations:** 1 Pharma Technical Development, F. Hoffmann-La Roche Ltd., Grenzacherstr. 124, CH-4070 Basel, Switzerland; 2 Center of Drug Absorption and Transport, Department of Biopharmaceutics and Pharmaceutical Technology, Institute of Pharmacy, University of Greifswald, Felix-Hausdorff-Str. 3, 17487 Greifswald, Germany; 3 Department of Psychiatry and Psychotherapy, University Medicine Greifswald, Ellernholzstr. 1/2, 17489 Greifswald, Germany; 4 Department of Internal Medicine and Geriatrics, University Medicine Greifswald, Ferdinand-Sauerbruch-Str., 17489 Greifswald, Germany

**Keywords:** solid oral dosage forms, swallowability, medicine acceptability, preference, older adults, geriatric patients

## Abstract

Older adults represent the major target population for oral medications, due to the high prevalence of multimorbidity. To allow for successful pharmacological treatments, patients need to adhere to their medication and, thus, patient-centric drug products with a high level of acceptability by the end users are needed. However, knowledge on the appropriate size and shape of solid oral dosage forms, as the most commonly used dosage forms in older adults, is still scarce. A randomized intervention study was performed including 52 older adults (65 to 94 years) and 52 young adults (19 to 36 years). Each participant swallowed four coated placebo tablets differing in weight (250 to 1000 mg) and shape (oval, round, oblong) in a blinded manner on three study days. The choice of tablet dimensions allowed for a systematic comparison between different tablet sizes of the same shape, as well as between different tablet shapes. Swallowability was assessed using a questionnaire-based method. All tested tablets were swallowed by ≥80% of adults, independent of age. However, only the 250 mg oval tablet was classified as *well swallowable* by ≥80% of old participants. The same was true for young participants; however, they also considered the 250 mg round and the 500 mg oval tablet as *well swallowable*. Furthermore, swallowability was seen to influence the willingness to take a tablet on a daily basis, especially for an intake over longer time periods.

## 1. Introduction

The global population of older adults (≥65 years) is continuously growing with an estimated increase from 9.6% up to 16.5% between 2020 and 2050 [1]. Despite the fact that the health status of older adults is heterogeneous, many of them are affected by multimorbidity. This means they are affected by two or more chronic conditions. Hence, older adults are often subject to polypharmacy, an intake of five or more medications. Therefore, they are the major group of medication end users. Health authorities issued a number of guidelines addressing the development of new drug products for this vulnerable patient group. Such guidelines define the need for safety and efficacy evaluations during clinical development in specific patient populations, as well as the development of age-appropriate formulations [2]. Similar guidelines have previously been released for the development of drug products intended for pediatric populations [3]. Even though both age categories represent vulnerable patient groups, development standards cannot simply be generalized as both groups differ in their abilities and impairments; therefore, their specific characteristics must be taken into account.

As many old adults are suffering from several chronic conditions, short- and especially long-term pharmacotherapy plays an important role for this patient population. A safe and effective therapy is only possible if patients adhere to their medication as intended [4,5]. The acceptability of a dosage form is key to patients’ adherence and is, therefore, a prerequisite for successful treatment. In the reflection paper on the pharmaceutical development of medicines for use in the older population, the European Medicines Agency, EMA, describes acceptability as the “ability and willingness of a patient to self-administer, and also of any of his/her lay or professional caregivers, to administer a medicinal product as intended” [2]. Consequently, characteristics of the dosage form as well as patients’ characteristics need to be considered in order to develop acceptable dosage forms. Designing pharmaceutical products with high acceptability by older patients contributes to patients’ quality of life, to healthy aging, and to the overall increase in life expectancy.

From a biological point of view, the natural consequence of aging is a continuous accumulation of molecular and cellular damages resulting in impaired organ and body functions [6]. This process can be accelerated by clinical conditions such as acute or chronic diseases. The resulting physiological, anatomical, and neurological alterations with advanced age can have a direct impact on the physical abilities of older adults. From an oral medication perspective, their ability to swallow can be reduced with increasing age and can, in the worst case, result in serious dysphagia [7,8,9].

Ruiz et al. assessed the preference of a range of paracetamol oral dosage forms in older adults. Powder for oral solution resulted to be the least preferred dosage form when compared to capsules and orodispersible tablets [10]. This suggests a certain preference towards solid oral dosage forms, SODFs, in contrast to liquid formulations in this population. SODFs provide key advantages over other dosage forms, including dose accuracy, stability, the potential combination of active ingredients, portability, and ease of administration [11]. Thus, capsules and especially tablets are widely used in older adults [11]. Besides swallowability, other influence factors on patients’ acceptability such as palatability, appearance, patient perception, and any handling to be conducted prior to use need to be considered according to the EMA reflection paper [2]. For SODFs, swallowability is not only important in terms of acceptability but also concerning their safe deglutition [8]. SODF characteristics such as size, shape, surface roughness, and taste have been reported to influence their swallowability and, hence, acceptability [12,13,14,15]. Furthermore, the disintegration time of SODFs and their tendency for swelling needs to be considered since they impact the mouthfeel and the ease of swallowing [15].

In general, data on acceptable size and shape of SODFs, specifically in older adults, are still scarce and heterogeneous. Miura et al. reported a tablet size of 7–8 mm as the preferred size to ensure good swallowability and handling in old, frail Japanese adults [16]. Vallet et al. found that tablets were accepted by old adults in general, but only tablets up to 6.5 mm in size were accepted by older adults with swallowing disorders [11]. In contrast, Liu et al. showed that SODFs of 11–13 mm (largest dimension) and 00-sized capsules were critical for swallowability in older adults with dysphagia [17]. In this study dysphagia was classified according to the Sydney Swallow Questionnaire which focuses on swallowing difficulties with liquids and foods, limiting its potential transferability on SODFs [17]. In terms of shape, most studies describe round tablets as the most preferred shape for smaller-sized tablets, while an elongated shape is preferred for larger-sized tablets [18,19].

So far, no standardized procedure for acceptability assessment has been established. Thus, different methods have been used in the past, which makes the comparison of study results difficult [20]. Further, acceptability has to be distinguished from patients’ preferences. While acceptability describes the ability and willingness of the patient and/or care giver to administer the product as intended, patients’ preferences provide relative comparison between products. However, the latter has limitations in terms of guiding the selection of the appropriate dosage form during pharmaceutical development [20,21]. As for the general acceptability, different approaches were used for the assessment of SODFs’ swallowability in the past. Some retrospective studies assessed swallowability according to participants’ experiences with their own medication [18,22]. In other studies, dosage forms needed to be swallowed by participants. The evaluation of swallowability was mainly performed via questionnaires, e.g., visual analogue scales were used by Hofmanová et al. [16,19,23,24,25]. Objective measurements were less frequently used for the evaluation of swallowability [26,27].

An appropriate size and shape is essential for the development of patient-centric SODFs. However, a knowledge gap in respect to suitable tablet sizes for older adults was described in the literature [20]. The present study aimed to systematically investigate the impact of size and shape on tablets’ swallowability in older adults and to compare it with the swallowability in young adults. We focused on tablets only, excluding capsules, to fully attribute any measured differences in swallowability to a difference in size or shape of the dosage forms. Tablets’ swallowability was assessed using a blinding method. Participants could not see the tablets before swallowing them in order to rule out any influence of visual perception on the assessment. Questionnaires were used to evaluate swallowability and its influence on patients’ willingness for a daily intake was inquired.

## 2. Materials and Methods

### 2.1. Manufacturing of Placebo Tablets

Placebo tablets of different shape and size, as detailed in Table 1, were manufactured by direct compression using a Riva Piccola B-D tablet press (Riva G.B. Ltd., Aldershot, UK). Tablet cores consisted of 76.5% microcrystalline cellulose (Avicel^®^ PH 102, DuPont Nutrition, Newark, DE, USA), 20.0% partially pregelatinized maize starch (Starch 1500^®^, Colorcon Inc., Harleysville, PA, USA), 0.5% hydrophilic fumed silica (Aerosil^®^ 200 Pharma, Evonik Resource Efficiency GmbH, Essen, Germany), and 3.0% sodium stearyl fumarate (PRUV^®^ Sodium Stearyl Fumarate, JRS Pharma GmbH & Co. KG, Rosenberg, Germany). The tablet cores were coated with a white polyvinyl alcohol-based coating (Opadry^®^ II Complete Film Coating System 85F18422 White, Colorcon Inc., USA) on a Glatt GMPC 1 Mini coater (Glatt, Binzen, Switzerland). The target tablet core weight and the target coating amount of 2.5 mg/cm^2^ was reached by ±5%. The consistent coating thickness for all tablets ensured constant surface characteristics during the deglutition process. Disintegration of the different tablets was recorded to be between 4.6 and 13.8 min.

The minimum cross-sectional area was calculated from the measured tablet dimensions (Table 1) based on its geometric shape.

### 2.2. Swallowability Assessment

For the swallowability assessment, 52 older adults (≥65 years) were randomly assigned to two experimental groups, Group 1 and Group 2. For the randomization, a block size of two and a stratification by sex (female vs. male) was applied, as female sex was reported to be a prognostic factor for self-reported swallowing difficulties with medication [18]. As a control to the old participants, the same was applied to younger adults (18 to 64 years; n = 52).

**Figure 1:**
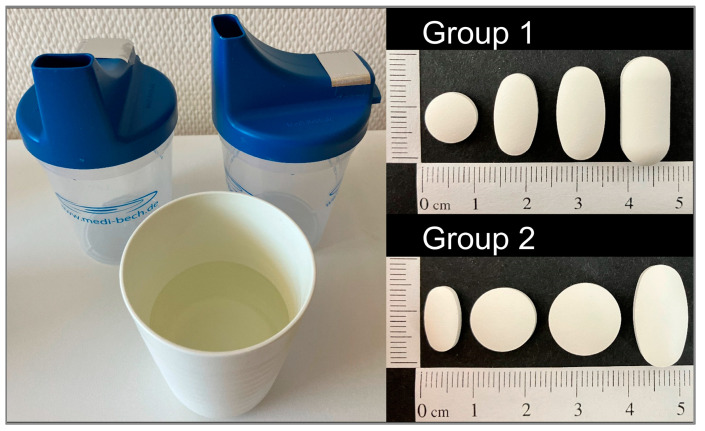
Medi-Bech containing one tablet at a time and cup of water provided with the four different, randomized tablets which were dependent on the group assignment.

Participants were asked to refrain from taking any medication, drinking, and eating up to one hour before the start of the assessment. Depending on the group assignment, participants were asked to swallow the same four tablets in a randomized order on three study days (Figure 1). Participants in Group 1 swallowed a 250 mg round, a 500 mg as well as a 750 mg oval, and a 1000 mg oblong tablet. Participants in Group 2 swallowed a 250 mg oval, a 500 mg as well as a 750 mg round, and a 1000 mg oval tablet. A time span of five minutes was kept between the deglutition of each of the four tablets. Tablets were provided to the participants in a Medi-Bech (Heller-Planung-Projektierung, Sankt Wolfgang, Germany), placed in the lid, on which the back hole was covered with aluminum foil (Figure 1). This blinding procedure ensured that participants did not see the tablets before swallowing them. In addition, a cup with 100 mL tap water at room temperature was provided. Participants were allowed to drink water ad libitum (max. 100 mL per tablet) and were asked to swallow the tablets in an upright position within one minute. If participants were not able to swallow any of the tablets, they were allowed to spit them out.

After having swallowed or having spit out the tablet, participants were asked to fill out the first part of a questionnaire (Supplementary Materials: Table S1, Figures S1 and S2). They needed to rate the swallowability (*not*, *moderately*, or *well swallowable*). If the tablet was rated as *not* or *moderately swallowable*, participants were asked for the reasons. Some possible reasons were given and could be ticked by participants (size, shape, stickiness, pain, and roughness), but any other reason could be stated. A further participant-reported outcome focused on foreign body sensation during the deglutition. Only if participants were able to swallow the tablet, they were further asked whether they bit on the tablet before swallowing it. In addition, they were queried about their willingness to take the tablet daily over either one week or several months. Researcher-reported outcomes included the time and water volume needed to swallow the individual tablets. Furthermore, the study team reported about any signs of aspiration, the number of attempts needed for the deglutition, and about participants’ facial expression (Supplementary Materials: Table S1, Figures S1 and S2).

### 2.3. Study Population

Participants were recruited between May and August 2022. Older participants were inpatients at the geriatric ward of the local hospital in Wolgast, Germany, Altersmedizinisches Zentrum Kreiskrankenhaus Wolgast gGmbH. Young participants were recruited via email and notices at the University of Greifswald, Germany. Each study participant signed an informed consent.

The inclusion criteria were age (≥65 years for the assessment in older adults; 18 to 64 years for the assessment in young adults) as well as a health status, enabling participation in the study. The health status of older adults was assessed at the Altersmedizinisches Zentrum Kreiskrankenhaus Wolgast gGmbH, Germany, by physicians, psychologists, and a speech therapist. Exclusion criteria were dementia, dysphagia, as well as cognitive impairments which would not allow participants to give informed consent and/or to follow the study instructions (old participants needed a score of ≥25 in the Mini-Mental State Examination). Other exclusion criteria were alcohol or drug dependency and self-reported eating disorders. For young participants, pregnancy and breast feeding were further exclusion criteria.

Demographic data of study participants were recorded, including: age, sex, body height and weight, presence of acute and/or chronic diseases, and current medications. All participants were asked if they managed their own medication, if existing, independently or if they were assisted. Furthermore, it was asked if participants have encountered difficulties when swallowing liquids or solids, foods, and/or oral medicines at present or in the past. For old participants, the results of the Mini-Mental State Examination and the Shulman clock drawing test, which were conducted as routine tests during hospitalization, were recorded [28,29]. In addition, results of logopedic assessments including results of the water swallowing test according to Daniels, if appropriate, were noted [30,31].

The study was approved by the Ethics Committee of the University Greifswald, Germany (internal registration number: BB 036/22).

### 2.4. Data Analysis

The swallowability of each tablet was rated three times (once on each of the three study days) by each participant. In order to summarize the three ratings into an overall category per participant and tablet, we used two different approaches. On the one hand, the ratings were summarized as: *not swallowable* vs. *swallowable*, to allow for a binary variable. If a tablet was swallowed on three out of three study days, it was categorized *swallowable*. If this was not the case, the tablet was categorized as *not swallowable*. On the other hand, for a stricter assessment of swallowability, a categorization into the binary variable of *not/moderately swallowable* vs. *well swallowable* was performed. Tablets were categorized as *well swallowable* if they were rated as *well swallowable* by the participant on three out of three study days. All other cases led to a tablet classification as *not/moderately swallowable*.

### 2.5. Statistical Analysis

A power analysis was performed using rpact 3.2.1—R Package for Adaptive Clinical Trials, based on the results of a pilot study (study unpublished). Comparing the rate of *not/moderately swallowable* vs. *well swallowable* for an uncoated 500 mg oval tablet of pi = 0.42 with an uncoated 1000 mg oval tablet of pi = 0.75 in twelve older adults resulted in an odds ratio of 0.24. Under the assumption of a one-sided significance level of α = 0.05 and a power of 80%, a sample size of 52 older participants (26 in each experimental group) was needed.

Demographic and general data on participants are given separately for the two age categories as mean ± standard deviation or as percentages.

**Figure 2:**
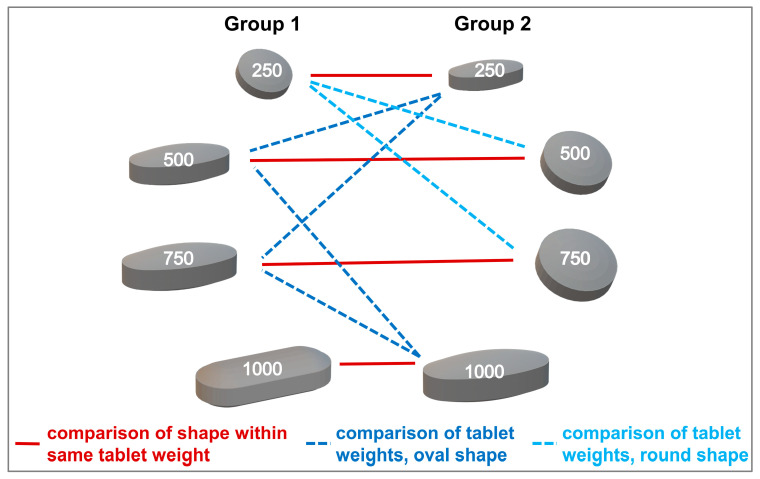
Comparison between tablets assigned to different experimental groups, applicable to both age categories.

Fisher’s exact test was used to compare binary swallowability ratings of different tablets within the same age category and to test the influence of age on the rating of tablets’ swallowability. For comparisons within the same age category, only tablets from the two different experimental groups (Group 1 or Group 2) were compared to each other in order to ensure comparison of independent data. The statistical test was done for tablets of same weight but differing in shape, and for tablets of same shape but differing in weight (Figure 2). To control the false discovery rate and account for multiple testing, *p*-values were corrected according to Benjamini and Hochberg [32]. If any adjusted *p*-values within a group of comparisons were <0.05, confidence intervals were also adjusted as proposed by Benjamini et al. [33]. In general, a *p*-value of <0.05 was deemed significant. Results are presented as *p*-values and odds ratios with their corresponding upper and lower confidence interval, CI, boundaries.

For the evaluation of time and amount of water needed to swallow a certain tablet, the mean was calculated from the three test occasions for each study participant. These participant-specific means were further summarized with median, mean ± standard deviation, minimum, and maximum for the individual tablets. This was done separately for the two age categories. Data analysis was performed with JMP^®^ 15.2.0 (466311) (SAS Institute Inc., Cary, NC, USA).

## 3. Results

### 3.1. Study Population

A total of 108 participants were randomized in the study, with 55 participants in the older population and 53 participants in the younger population. There were three dropouts among the old participants and one amongst the young participants. Dropouts were due to early hospital discharge, nausea (not caused by the study participation), or non-compliance. Hence, 52 participants were finally included in each age category. In both age groups, 25 participants were assigned to Group 1 and 27 participants were assigned to Group 2.

The demographic data of the study population is summarized in Table 2. The old population was 81.3 ± 7.3 years old and included youngest (65–74 years), middle (75–84 years), and oldest (≥85 years) old adults [34]. The young population represented young adults with a mean age of 23.1 ± 3.8 years. The number of female participants was higher in the old compared to the young population. A total of 98.1% of old participants were affected by two or more chronic conditions. Further, 96.2% of old participants were regularly taking five or more medications. More than half of the old population (51.9%) was treated with ten or more medications. A total of 46.2% of old participants were managing the preparation and administration of their daily medication independently. The other old participants were assisted either by family members, mobile nursing services, or caregivers at retirement homes. No young participant reported swallowing difficulties with medication at present, but 21.2% did report having been affected by such difficulties in the past. However, 15.4% of old participants reported swallowing difficulties with medication at present and in the past, while an additional 9.6% were only affected in the past. Thus, 75.0% of old participants and 78.9% of young participants never had swallowing difficulties with medication. In respect to the deglutition of liquids or solid foods, 9.6% and 7.8% of old participants, respectively, reported to be or having been affected by swallowing difficulties at present or in the past. No young participant reported having difficulties with swallowing foods or drinks.

All old participants showed a score of 25 or higher in the Mini-Mental State Examination which implicates no or mild cognitive impairment. Further, they all scored three or lower (out of six) in the Shulman clock drawing test, which further confirmed no or only mild cognitive impairments among the old study population. For three participants (5.7%), no clock drawing test score was available as they were not able to write when the test was scheduled. The results of logopedic assessments, including the water swallowing test according to Daniels, confirmed that there was no diagnosable swallowing difficulty in any of the participants. Thus, these tests affirmed safe participation and sufficient cognitive capability for the old participants to be included in the tablets’ swallowability assessment.

### 3.2. Swallowability Assessment

**Figure 3:**
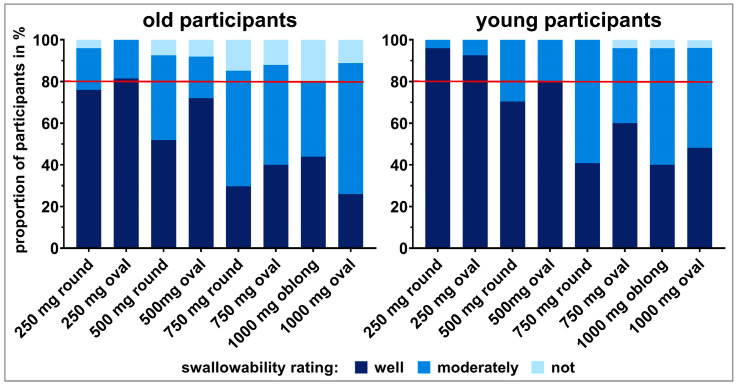
Summary of swallowability ratings (*not swallowable*, *moderately swallowable*, and *well swallowable)* for each of the tablets tested in old and young participants. The rating of each participant comprises the swallowability evaluations of three study days. Taking the ratings *not* and *moderately swallowable* together and comparing those to the ratings *well swallowable* allows for a strict qualitative swallowability assessment. However, when taking the two ratings *well* and *moderately swallowable* together and comparing to the rating *not swallowable*, swallowability capabilities can be evaluated. The red line represents 80% of participants.

**Figure 4:**
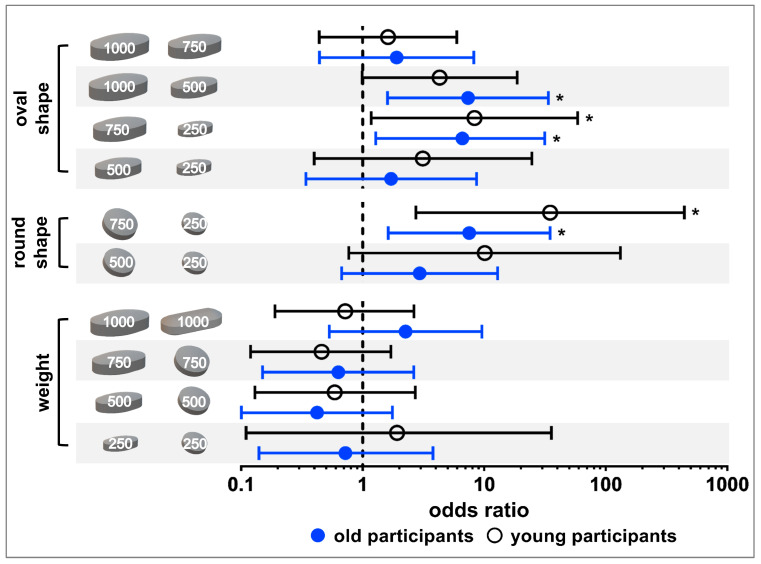
Statistical analysis of tablet ratings categorized as *well swallowable* vs. *not/moderately swallowable* using the Fisher’s exact test and applying Benjamini Hochberg correction. Results for old participants are shown in blue, while those for young participants are shown in black. Dots and circles are representing odds ratios and bars are marking the upper and lower confidence interval boundaries (98.5% confidence interval for old participants and 98.0% confidence interval for young participants). Asterisks indicate significant difference.

The tablet ratings were dichotomized into *well swallowable* vs. *not/moderately swallowable* for a strict qualitative assessment of swallowability (Section 2.4.; raw data is shown in the Supplementary Materials Figure S3). A cut off value was defined at 80% of participants as this is a commonly used threshold for acceptability [10]. Only the 250 mg oval tablet was classified as *well swallowable* by ≥80% of old participants. Young participants considered the 250 mg tablets, independent of their shape, as well as the 500 mg oval tablet as *well swallowable* (Figure 3). In general, with increasing size of same shaped tablets there was a trend towards a decrease in the percentage of *well swallowable*. In both age groups, a significant difference in swallowability was seen between tablets of the same shape with a weight difference of 500 mg (i.e., 250 mg vs. 750 mg round tablets and 250 mg vs. 750 mg oval tablets), according to the Fisher’s exact test and after Benjamini Hochberg correction (Figure 4; Supplementary Materials: Tables S2 and S3). For old participants, this further held true for the 500 mg vs. 1000 mg oval tablets. However, for young participants the comparison between the 500 mg vs. 1000 mg oval tablets, as well as between the 250 mg and 500 mg round tablets, did boarder statistical significance. When comparing tablets of the same weight, the smaller the minimum cross-sectional area, the more frequently a tablet was classified as *well swallowable*. An exception was only observed with the 250 and 1000 mg tablets in young adults. In terms of differences between age categories, no significant difference was observed, when comparing the swallowability rating of each individual tablet in young vs. old participants (Supplementary Materials: Table S4).

Summarizing the three swallowability ratings of each participant for the different tablets into *swallowable* and *not swallowable* resulted in a classification of tablets according to participants’ swallowing capabilities (Figure 3). All tablets could be swallowed by ≥80% of participants independent of their age. Nevertheless, except for the 250 mg oval tablet, there were always some old adults (4–20%) who could not swallow individual tablets, independent of size and shape. The proportion of older adults not being able to swallow the tablets increased with increasing tablet size (Figure 3). A different observation was done in young participants. All young participants were able to swallow tablets up to 500 mg, as well as the 750 mg round tablet. Less than 5% of young participants were not able to swallow the 750 mg oval tablet, as well as tablets of 1000 mg weight. However, according to Fischer’s exact test and after Benjamini Hochberg correction this classification of swallowability did not result in statistical difference for the different tablets, neither within age categories nor between age categories (Supplementary Materials: Tables S5–S7).

#### 3.2.1. Acceptability of Tablets for Short- and Long-Term Intake

**Figure 5:**
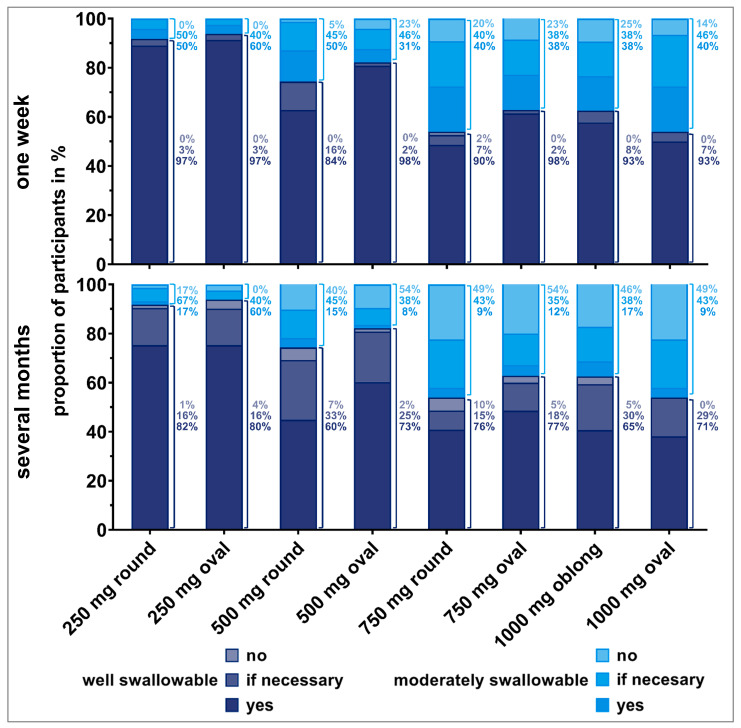
Willingness (*no*, *if necessary*, *yes*) of old participants to swallow the respective tablet once a day for one week (upper panel) or over several months (lower panel), dependent on tablet’s swallowability rating. Only the answers from participants who were able to swallow the tablets were considered here (corresponding to 100% value). Thus, zero to three evaluations from each participant are included. Percentages to the right of the bars are representing the fractions of the different willingness levels, if all deglutition attempts of each swallowability rating (*well* or *moderately swallowable*) are set to 100%.

**Figure 6:**
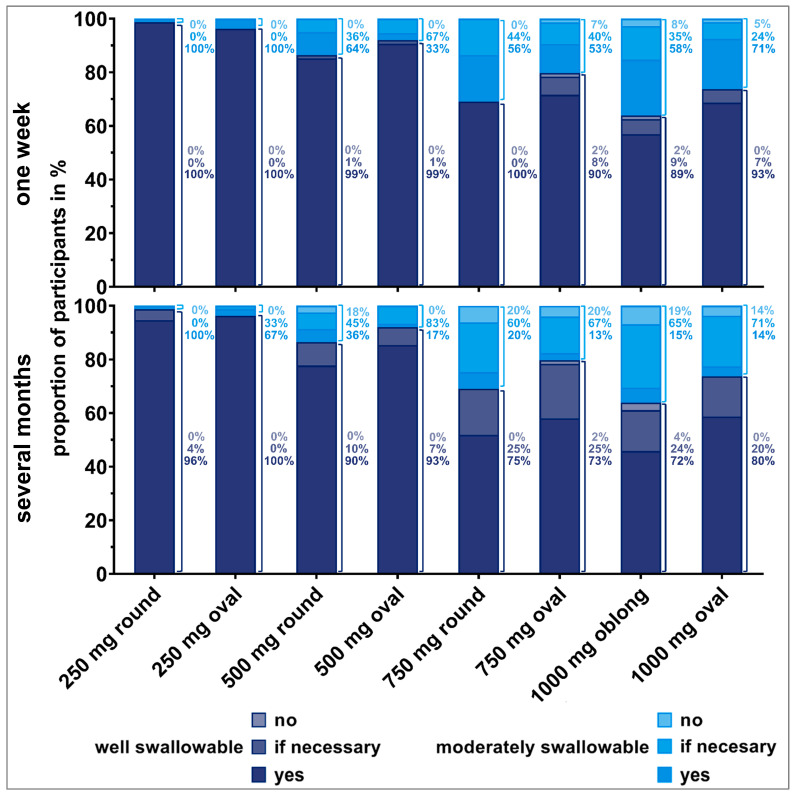
Willingness (*no*, *if necessary*, *yes*) of young participants to swallow the respective tablet once a day for one week (upper panel) or over several months (lower panel) dependent on tablet’s swallowability rating. Only the answers from participants who were able to swallow the tablets were considered here (corresponding to 100% value). Thus, zero to three evaluations from each participant are included. Percentages to the right of the bars are representing the fractions of the different willingness levels, if all deglutition attempts of each swallowability rating (*well* or *moderately swallowable*) are set to 100%.

Each time participants were able to swallow an individual tablet, they were asked if they would be willing to swallow the tablet once daily over (i) one week or (ii) over several months. Figure 5 and Figure 6 show the resulting answers (*no*, *if really necessary*, *yes*) by old and young participants, respectively. In order to put their willingness to swallow a tablet in relation to their ability to swallow the same tablet, the data were stratified by the swallowability rating. Within each bar, the 100% value represents all successful deglutition attempts that were rated as *well* or *moderately swallowable*. Within the bars, percentages for the different willingness ratings (*no*, *if really necessary,* or *yes*) are shown, dependent on the swallowability rating given for the corresponding deglutition. Percentages to the right of the bars are representing the fractions of the different willingness levels, if all deglutition attempts of each swallowability rating (*well* or *moderately swallowable*) are set to 100%.

We observed an influence of participants’ deglutition experience on their willingness to take a dosage form over a short or long period of time. The willingness clearly decreased if the tablet needed to be taken over several months compared to a short treatment period, especially for old participants. Young participants seemed to be more willing to take even larger tablets over a long period of time. The influence of the number of regular medications on the willingness to take a medication short- and long-term revealed no clear trend.

More than 80% of participants who rated the tablets as *well swallowable* would be willing to take the corresponding tablet once daily for one week. This finding was independent of participants’ age. However, the willingness decreased if participants reported tablets to be *moderately swallowable*. For instance, for the 500 mg oval tablet, 98% of old participants who considered the tablet as *well swallowable* would be willing to take it once daily for a week. However, this was the case for only 31% of old participants if they stated that the tablet was *moderately swallowable*, and 23% of them were even rejecting a daily intake. Similar observations were made for the 750 mg round and oval tablets, as well as for the 1000 mg oblong tablet. More than 20% of old participants, having rated those tablets as *moderately swallowable*, would not be willing to take these tablets short-term.

Over 80% of old participants who rated the 250 mg round and oval tablets as *well swallowable* were willing to take those tablets daily over several months. However, willingness was limited for tablets exceeding a weight of 250 mg, even if they were rated as *well swallowable.* Young participants were willing to take tablets up to 500 mg of weight daily over several months in cases they were rated as *well swallowable*. Young participants’ willingness was slightly reduced for tablets of 750 mg or higher weight, even if rated as *well swallowable*. If tablets’ swallowability was rated as *moderate*, over 80% of young participants would be willing to take those tablets daily for a long-term treatment. However, some of those young participants would only be willing if the intake was really necessary.

#### 3.2.2. Reasons for Poor Swallowability

**Figure 7:**
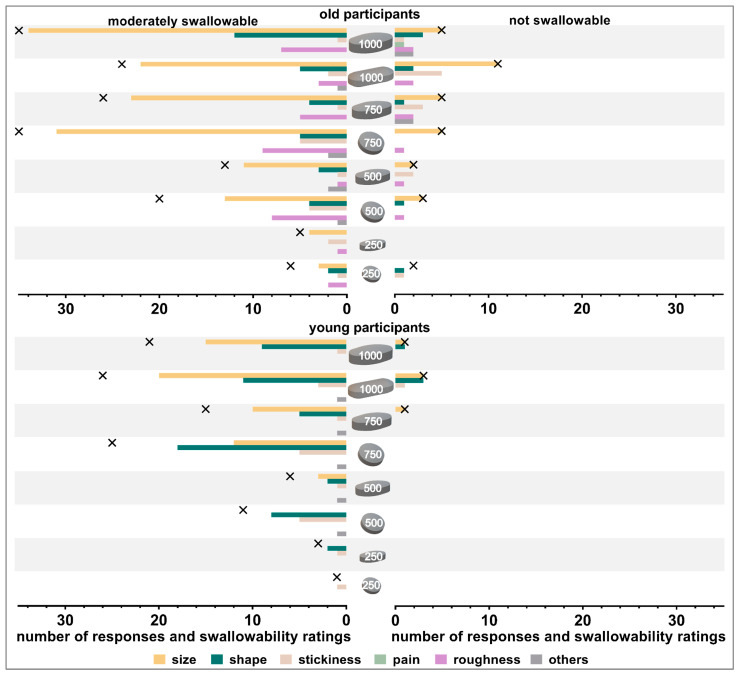
Number of responses and swallowability ratings (*moderately swallowable* shown on left panels and *not swallowable* shown on right panels). Crosses indicate the number of swallowability ratings (absolute numbers). Data are shown separately for old participants (upper panel) and young participants (lower panel).

Participants who rated a tablet as *moderately* or *not swallowable* were asked for the specific reason leading to the poor swallowability rating. Size was the most frequent reason reported by old participants (Figure 7), while for young participants shape and size were frequently stated reasons. The tablet shape was mentioned more often in cases of large round compared to oval tablets (500 and 750 mg in weight). Although the same coating type and amount was applied on all tablets, old participants reported that tablet roughness was a reason for moderate swallowability of some tablets, especially those with a minimal cross-sectional area of >45 mm^2^. Stickiness of tablets was rarely reported as a reason, independent of tablet size and shape and participants’ age. Other reasons reported by old participants included: tablet was too edged, tablet lay vertically in the mouth, tablet caused immediate urge to gag, and tablet was dull/sandy and therefore tablet’s gliding was impaired. Besides size, shape, and stickiness, tablet’s surface, as well as tablet’s positioning in participants’ mouth caused impaired swallowability for young participants.

#### 3.2.3. Characteristics Describing the Deglutition Process

**Figure 8:**
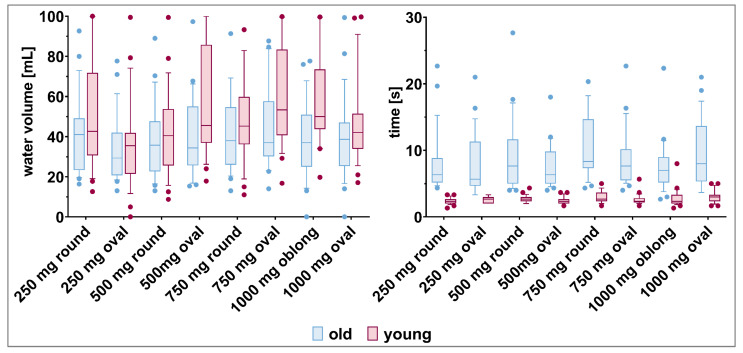
Water volume (left panel) and time (right panel) needed by old and young participants to swallow the tablets (mean, 10th, 90th percentile, and outliers are shown).

*Water consumption.* The volume of water used to swallow the different tablets was highly variable for the individual participants, irrespective of their age (Figure 8, Supplementary Materials: Table S8). Old participants tended to use less water to swallow the tablets compared to young participants (34.5–44.6 mL vs. 37.2–58.6 mL, respectively).

After removing the cup of water from the mouth for the first time, study participants sometimes drank some more water. Compared to young participants, old participants brought the cup again to their mouth more often to drink additional water (Table 3). However, while no clear association was seen with tables’ size and shape in older participants, young participants tended to drink additional water more often with increasing tablet size.

*Time needed for deglutition.* In terms of time needed to swallow the different tablets, young participants were faster compared to old participants (Figure 8). It took the young participants only two to three seconds to swallow the different tablets. In contrast, old participants swallowed the tablets, on average, within seven to eleven seconds, but with high variability. The tablet size and shape did not influence the deglutition time.

*Biting on tablet.* Neither old nor young participants bit on any of the tablets before swallowing them. If tablets could be swallowed, they were swallowed whole. Otherwise, the tablets were spit out.

*Foreign body sensation.* Compared to old participants, young participants more often reported a foreign body sensation during the attempt to swallow the different tablets, as well as during the actual deglutition (Table 4). Round tablets caused a foreign body sensation more often compared to oval shaped tablets of same weight.

*Signs of aspiration.* In only ≤10% of cases, signs of aspiration were reported independent of participants’ age (Supplementary Materials: Figure S4). When signs of aspiration occurred, participants most often cleared their throat. For old participants this occurred most often when swallowing the 250 mg oval tablet while for young participants it occurred most often with the 1000 mg oblong tablet. Overall, no association with tablet size nor shape was seen.

*Number of swallowing attempts and facial expression.* Neither the observation of number of attempts needed to swallow the different tablets nor the one of facial expression were used for further swallowability evaluations. Both observations were biased by observers and, therefore, were neither sufficiently objective nor comparable.

## 4. Discussion

In this study, we systematically investigated the influence of tablet size and tablet shape on swallowability in older adults, as the main medication end users, and in young adults as a control group.

Older adults are a heterogeneous patient population; therefore, all of their characteristics, capabilities, and impairments need to be considered. The vast majority of old participants (98.1%) were affected by two or more chronic conditions and, consequently, polypharmacy was very common (96.2% polypharmacy, 51.9% hyperpolypharmacy). These numbers for (hyper-) polypharmacy are higher compared to values for old adults stated in a number of literature references [35,36,37,38]. However, they are in line with numbers being reported for participants taking part in a study using the ClinSearch Acceptability Score Test^®^ conducted by Vallet et al. [39]. The rather vulnerable profile of included old participants might be due to the recruitment site being a hospital, thereby including sicker people compared to the general population [40]. As older adults are commonly dependent on the intake of medicines, these rather vulnerable participants are a good point of reference.

Depending on the setting, the prevalence of dysphagia within older adults was reported to be 23%, 44%, and up to 50% within independently living older adults, patients admitted to acute geriatric units, or older adults living in nursing homes, respectively [41]. However, patients suffering from dysphagia were excluded from this study for safety reasons. Aside from dysphagia, the prevalence of self-reported swallowing difficulties with medication was reported to be between 14% and 40% in the general population [18,42,43,44]. Schiele et al. stated a prevalence of 37.4% on average for adults with a mean age of 61.8 ± 15.6 years, but even higher prevalence for young female participants. In contrast, no young participant in our study reported swallowing difficulties with medication at present, and less than a quarter have had experienced such difficulties in the past. Female sex was not associated with a higher prevalence in swallowing difficulties with medication. The different recruitment sites being general practices in contrast to the university might have caused this difference. However, the rather young average age of young participants implies an inclusion of those young adults being most often affected by swallowing difficulties with medication, according to Schiele et al. [18]. Thus, swallowing capabilities of younger adults are unlikely to have been overestimated in this study.

The size of a SODF describes its dimensions. It is characterized by two independent parameters: the shape and the weight of the individual dosage form. However, some previous studies compared tablets’ size with each other by using the largest dimension, neglecting their shape and different tablet weights. Thus, as previously discussed, tablets’ minimum cross-sectional area together with shape should be considered for comparisons within same weight tablets [18]. In order to test sizes and shapes of tablets that old adults are commonly confronted with in daily life, we undertook orientation of the 300 most prescribed drug products being prescribed by independent physicians to outpatients, 65 years or older. Those drug products were dispensed by public or hospital pharmacies to the account of statutory health insurances in Germany, during the data year 2020 (data source: GKV-Arzneimittelindex im Wissenschaftlichen Institut der AOK). A total of 84.3% of those drug products accounted for SODFs that need to be swallowed whole. Hereof, 94.1% accounted for tablets and 5.9% for capsules. Thus, by focusing on tablets the majority of most commonly prescribed dosage forms were covered and no difference in dosage forms’ density needed to be considered [45]. For the design of all tablet shapes (round, oval, and oblong), we followed the dimensions of these 300 most prescribed drug products (Supplementary Materials: Figures S5 and S6). The value of 250 mg was taken as the lower limit for tablets’ weight, as tablets being <6.5 mm were reported to be even accepted by older adults with swallowing disorders [11]. By applying the same coating to all tablets, swallowability could be assessed under constant conditions. Thus, bias of different surface characteristics and different palatability were kept to a minimum.

When thinking about patients’ daily medication intake, deploying blinding to tablets’ deglutition may occur as an unrealistic scenario. However, this approach allowed to study participants’ actual swallowability capabilities. It enabled exclusion of the strong bias of visual perception and, thus, difficulties in swallowability due to psychological aspects [46]. It was observed that there exists a certain variability in swallowability dependent on participants’ form of the day (unpublished data). Therefore, this variability was considered by repeating the deglutition on three study days with each participant. Moreover, the deglutition of too many tablets after each other is likely to bias swallowability evaluation. Thus, a number of four tablets was deemed appropriate. However, we wanted to test more than four tablets and, therefore, the eight tablets were assigned to two subgroups. Arrangement of the different tablets was performed to allow for the most interesting comparisons of tablets using independent data. Further, difficulties in utilizing a visual analogue scale by old participants were observed in a pilot study (unpublished data). Therefore, we used a simple swallowability rating in three categories, hazarding a possible loss in discriminatory power between tablets’ swallowability.

Even if tablets, which were up to 1000 mg in weight and differed in shape were swallowed by ≥80% of old and young participants, we generally observed more difficulties in swallowing as the size of the individual tablets increased. This trend was seen independent of tablets’ shape, in both age categories, and is in line with previous findings [19]. However, good swallowability was only reached by tablets of lower weight. In general, some differences were identified between old and young participants. The decreased swallowability in older participants is in accordance with the generally observed decline in swallowing capabilities with increasing age [8,9]. Further, the fact that tablets up to 1000 mg in weight were swallowed by over 80% of old participants where blinding was used might indicate that, besides decreased swallowing capabilities due to increasing age, an age-independent psychological barrier may be rather high. This psychogenic dysphagia was already described earlier as phagophobia [46]. Sex did not seem to have an influence within old participants, while young female participants tended to state worse swallowability compared to male young participants.

We observed that, for old participants within the 250 mg tablets and for young participants within the 500 mg tablets, the oval shape and not the round one was still *well swallowable* by over 80% of participants. This is in accordance with earlier studies demonstrating that with increasing size of tablets an elongated shape is preferred over a round shape [17,18,19]. This preference also confirms that the minimum cross-sectional area, rather than the largest tablet dimension, should be used when comparing differently shaped tablets, as recommended by Schiele et al. [18].

A comparison of our results with those in the literature is shown in Table 5. Based on the different in- and exclusion criteria, as well as different methodological approaches that were used [20], a direct comparison is rather difficult. Only the study conducted by Miura et al. included an actual deglutition of dosage forms, as it was done in our study [16]. Compared to acceptable tablet sizes reported by Miura et al. [16], our study resulted in slightly higher size limits. However, our results on *well swallowable* tablets for old adults were comparable to the SODF sizes Schiele et al. reported not to cause swallowing difficulties in adult patients of general practices, as well as those reported by Liu et al. for older adults with dysphagia [17,18].

Even if 150 mL or 240 mL are recommended by EMA or the U.S. Food & Drug Agency, respectively, we provided only 100 mL of water for the deglutition of the different tablets [47,48]. The reason was to keep the bias on swallowability evaluations of drunk water to a minimum. However, on average, participants used less than 60 mL of water to swallow the different tablets. Thus, participants used considerably less water for the deglutition than is generally recommended. Moreover, old participants tended to use less water compared to young participants. Gallo et al. reported a positive association of successful tablet deglutition with the volume of water being used [49]. This is in line with the lower number of successful deglutitions observed for old compared to young participants in our study. In addition to the slightly higher used water volume, young participants took the cup less often down from their mouth. Thus, we assume that young participants took larger gulps, which was beneficial for successful and fast deglutition. Further, old participants needed more time to swallow the different tablets. This is in line with observations made by Hofmanová et al. [23].

In accordance with previous findings, we observed that tablet shape and size were related to the occurrence of swallowing difficulties [14,18]. The influence of size seemed to be of major importance compared to the one of shape. Thus, one should focus on the minimum cross-sectional area when comparing different tablets. The application of coatings can influence surface properties that are unfavorable for swallowing; however, coatings may have less impact on swallowability than tablet size or shape. Even though surface characteristics were the same for all tablets in this study, the surface was described as sticky, rough, or sandy, probably impairing swallowability for some participants. Surprisingly, surface roughness was stated more often by old participants, even if one would expect decreased sensory abilities for older adults compared to their younger counterparts [50]. The higher perception of surface roughness within old participants might be caused by a reduced amount of saliva, resulting in reduced lubrication [51,52]. Reducing surface roughness, by applying improved coatings, might improve tablets’ swallowability from *moderate* to *good*. The surface characteristics seemed to be of greater importance as tablets’ minimum cross-sectional area was increasing, resulting in higher contact areas between tablet and mucosa [53]. In contrast, the lower frequency of foreign body sensation in older adults compared to younger adults is in accordance with the assumption of reduced sensory abilities with increasing age.Besides, older adults might be more trained in taking oral medications, which could result in a higher tolerability towards foreign body sensation. This is further supported by a previous finding that younger adults are more often affected by swallowing difficulties with medication, most likely caused by psychological aspects [18,43,54].

The influence of swallowability on the overall acceptability of tablets was studied by asking participants about their willingness to take the specific tablet once daily over a short and long period of time. The treatment duration (i.e., short- vs. long-term treatment), seemed to be more important for old compared to young adults. No association between the number of regular medication taken by old participants and their willingness for a daily tablet intake was observed. However, a generally higher daily pill burden of older adults compared to the one of younger adults could cause the difference between the age categories. Thus, it strengthens the need to consider patients with all their conditions in order to develop patient-centric drug products [2,5]. Our results suggest that moderate swallowability might be sufficient for short term treatments, such as for one week. However, in developing an acceptable dosage form that a patient will adhere to during long-term treatment, good swallowability has to be provided. This should be considered particularly in the vulnerable patient population of older adults.

## 5. Conclusions

Blinded deglutition testing showed that tablets up to 1000 mg were swallowable for over 80% of adults, independent of their age. However, when applying a stricter qualitative swallowability classification, size limits were distinctly lower. Only the lightest tested tablet exhibiting a weight of 250 mg and being of oval shape was categorized as *well swallowable* for over 80% of old participants. For young participants, this held true for tablets of 250 mg weight, independent of their shape, as well as for a 500 mg oval tablet. Moreover, tablets’ swallowability was observed to influence the willingness and, thus, the overarching acceptability of tablets’ daily intake over different time periods, be it one week or several months.

Based on these results, the study provides tablet size limits according to deglutition capabilities, excluding the bias of visual perception. An age dependency was observed which needs to be respected during patient-centric drug development, especially for the vulnerable patient population of older adults. Increasing acceptability by providing appropriate tablet sizes and shapes might support patients’ adherence.

## Figures and Tables

**Table 1:** Description of tablets’ characteristics. The group assignment refers to the experimental groups in the deglutition assessment (Section 2.2).

Shape	Weight [mg]	Diameter [mm]	Cap Radius [mm]	Thickness [mm]	Min. Cross Sectional Area [mm^2^]	Group
round	250	9.30	11.60	3.82	29.63	1
round	500	11.70	14.71	4.82	47.13	2
round	750	13.35	16.86	5.47	60.94	2
**Shape**	**Weight [mg]**	**Length** **[mm]**	**Width [mm]**	**Thickness [mm]**	**Min. Cross Sectional Area [mm^2^]**	**Group**
oval	250	12.00	6.25	4.24	22.17	2
oval	500	15.10	7.87	5.31	35.15	1
oval	750	17.30	9.02	6.12	46.12	1
oval	1000	19.00	9.90	6.66	55.64	2
oblong	1000	20.50	8.89	6.07	44.24	1

**Table 2:** Characterization of participants, subdivided in the two age categories: old and young.

	Old Participants	Young Participants
Number of participants	52	52
Female	67.3%	55.8%
Age (mean ± SD)	81.3 ± 7.3 years	23.1 ± 3.8 years
65 to 74 years old	23.1%	-
75 to 84 years old	42.3%	-
85 years or older	34.6%	-
BMI ^1^ (mean ± SD)	28.5 ± 7.4 kg/m^2^	23.8 ± 3.5 kg/m^2^
Number of regular medications (mean ± SD) ^2^	9.7 ± 3.1	0.3 ± 0.5

^1^ Body Mass Index; ^2^ On-demand medication was excluded from this evaluation.

**Table 3:** Repeated water intake after the cup of water has been removed from the mouth for the first time. Data are shown separately for the two age categories and include three observations per study participant for each tablet.

Tablet	Proportion of Participants in %					
	Old Participants			Young Participants		
	No	Yes, Once	Yes, Multiple Times	No	Yes, Once	Yes, Multiple Times
250 mg round	32.0	17.3	50.7	81.3	6.7	12.0
250 mg oval	39.5	13.6	46.9	87.7	3.7	8.6
500 mg round	33.3	24.7	42.0	77.8	2.5	19.8
500 mg oval	37.3	17.3	45.3	86.7	8.0	5.3
750 mg round	29.6	33.3	37.0	70.4	12.3	17.3
750 mg oval	28.0	18.7	53.3	80.0	8.0	12.0
1000 mg oval	34.6	22.2	43.2	67.9	7.4	24.7
1000 mg oblong	38.7	14.7	46.7	70.7	13.3	16.0

**Table 4:** Foreign body sensation caused by tablets during swallowing. Data are shown separately for young and old participants including three evaluations per study participant for each tablet.

Tablet	Proportion of Participants in %					
	Old Participants			Young Participants		
	No	Yes, during Attempt	Yes, during Deglutition	No	Yes, during Attempt	Yes, during Deglutition
250 mg round	96.0	0.0	4.0	88.0	0.0	12.0
250 mg oval	92.6	1.2	6.2	95.1	0.0	4.9
500 mg round	81.5	0.0	18.5	72.8	0.0	27.2
500 mg oval	93.3	1.3	5.3	78.7	0.0	21.3
750 mg round	63.0	0.0	37.0	58.0	0.0	42.0
750 mg oval	85.3	5.3	9.3	68.0	1.3	30.7
1000 mg oval	74.1	2.5	23.5	63.0	1.2	35.8
1000 mg oblong	85.3	6.7	8.0	57.3	4.0	38.7

**Table 5:** Studies focused on tablet sizes and tablet shapes associated with swallowing difficulties in (older) adults.

Studied Population	Study Type	Tested Dosage Forms	Acceptable SODF Size ^1^	
Frail older Japanese persons, n = 73	Deglutition of different tablets	Different tablets (unknown size) ^2^	Tablets ≤ 7–8 mm	[16]
Older adults, mean age of 74 years, n = 156	Anticipated swallowability according to visual perception	Tablets: round and elongated shapes of 5–13 mm (d or l); hard gelatin capsules: size 4 to 00	Older adults with swallowing difficulties (11% of study population): tablet sizes <11–13 mm (d or l) and capsules of size 0 or smaller	[17]
Adult patients, mean age of 61.8 ± 15.6 years, n = 1051	Patients reported swallowing difficulties among their own regular medications.	Retrospective analysis of patients’ regular medications	Round tablets ≤8.1 mm (d) and ≤3.5 mm (h); oval tablets ≤13.2 mm (l), ≤6.6 mm (w), and ≤4.6 mm (h); oblong tablets ≤13.3 mm (l), ≤6.2 mm (w), and ≤4.9 mm (h)	[18]
Older adults, mean age 81.3 ± 7.3 years, n = 52	Deglutition of different tablets	Round tablets: 9.30–13.35 mm (d); oval tablets: 12.00–19.00 mm (l); oblong tablet: 20.50 mm (l)	*Well swallowable*: oval tablet 12.00 mm (l); *Swallowable*: round tablets ≤13.35 mm (d), oval tablets ≤19.00 mm (l), oblong tablet 20.50 mm (l)	Present study
Younger adults, mean age 23.1 ± 3.8 years, n = 52			*Well swallowable*: round tablet 9.30 mm (d), oval tablets ≤15.10 mm (l); *Swallowable*: round tablets ≤13.35 mm (d), oval tablets ≤19.00 mm (l), oblong tablet 20.50 mm (l)	

^1^ Dosage forms’ dimensions are described in this table by d = diameter, l = length, w = width, and/or h = height. ^2^ Only abstract available in English language.

## Supplementary Materials

The following supporting information can be downloaded at: https://www.mdpi.com/article/10.3390/pharmaceutics15041042/s1.

## References

[1] U.N. World Population Prospects 2022. https://population.un.org/wpp/Download/Standard/MostUsed/.

[2] EMA Reflection Paper on the Pharmaceutical Development of Medicines for Use in the Older Population. https://www.ema.europa.eu/en/documents/scientific-guideline/reflection-paper-pharmaceutical-development-medicines-use-older-population-first-version_en.pdf.

[3] EMA Guideline on Pharmaceutical Development of Medicines for Paediatric Use. https://www.ema.europa.eu/en/documents/scientific-guideline/guideline-pharmaceutical-development-medicines-paediatric-use_en.pdf.

[4] Stegemann S., Ternik R.L., Onder G., Khan M.A., van Riet-Nales D.A. (2016). Defining Patient Centric Pharmaceutical Drug Product Design. Aaps J..

[5] Stegemann S., Sheehan L., Rossi A., Barrett A., Paudel A., Crean A., Ruiz F., Bresciani M., Liu F., Shariff Z. (2022). Rational and Practical Considerations to Guide a Target Product Profile for Patient-Centric Drug Product Development with Measurable Patient Outcomes—A Proposed Roadmap. Eur. J. Pharm. Biopharm..

[6] Roller-Wirnsberger R., Thurner B., Pucher C., Lindner S., Wirnsberger G.H. (2020). The Clinical and Therapeutic Challenge of Treating Older Patients in Clinical Practice. Br. J. Clin. Pharmacol..

[7] Stegemann S., Gosch M., Breitkreutz J. (2012). Swallowing Dysfunction and Dysphagia Is an Unrecognized Challenge for Oral Drug Therapy. Int. J. Pharm..

[8] Liu F., Ranmal S., Batchelor H.K., Orlu-Gul M., Ernest T.B., Thomas I.W., Flanagan T., Tuleu C. (2014). Patient-Centered Pharmaceutical Design to Improve Acceptability of Medicines: Similarities and Differences in Paediatric and Geriatric Populations. Drugs.

[9] Perrie Y., Badhan R.K.S., Kirby D.J., Lowry D., Mohammed A.R., Ouyang D. (2012). The Impact of Ageing on the Barriers to Drug Delivery. J. Control. Release.

[10] Ruiz F., Vallet T., Wojcicki A.D., Belissa É., Fontan J.-E., de Pontual L., Nathanson S., Chevallier A., Laribe-Caget S., Boudy V. (2019). Dosage Form Suitability in Vulnerable Populations: A Focus on Paracetamol Acceptability from Infants to Centenarians. PLoS ONE.

[11] Vallet T., Michelon H., Orlu M., Jani Y., Leglise P., Laribe-Caget S., Piccoli M., Fur A.L., Liu F., Ruiz F. (2020). Acceptability in the Older Population: The Importance of an Appropriate Tablet Size. Pharm.

[12] Shariff Z., Kirby D., Missaghi S., Rajabi-Siahboomi A., Maidment I. (2020). Patient-Centric Medicine Design: Key Characteristics of Oral Solid Dosage Forms That Improve Adherence and Acceptance in Older People. Pharm.

[13] Notenboom K., Leufkens H.G., Vromans H., Bouvy M.L. (2017). Learning from Patients: Identifying Design Features of Medicines That Cause Medication Use Problems. Int. J. Pharm..

[14] Andersen O., Zweidorff O., Hjelde T., Rødland E. (1995). Problems When Swallowing Tablets. A Questionnaire Study from General Practice. Tidsskr. Den Nor. Laegeforening Tidsskr. Prakt. Med..

[15] Drumond N., Stegemann S. (2020). Better Medicines for Older Patients: Considerations between Patient Characteristics and Solid Oral Dosage Form Designs to Improve Swallowing Experience. Pharm.

[16] Miura H., Kariyasu M. (2007). Effect of Size of Tablets on Easiness of Swallowing and Handling among the Frail Elderly. Nippon Ronen Igakkai Zasshi Jpn. J. Geriatr..

[17] Liu F., Ghaffur A., Bains J., Hamdy S. (2016). Acceptability of Oral Solid Medicines in Older Adults with and without Dysphagia: A Nested Pilot Validation Questionnaire Based Observational Study. Int. J. Pharm..

[18] Schiele J.T., Quinzler R., Klimm H.-D., Pruszydlo M.G., Haefeli W.E. (2013). Difficulties Swallowing Solid Oral Dosage Forms in a General Practice Population: Prevalence, Causes, and Relationship to Dosage Forms. Eur. J. Clin. Pharmacol..

[19] Overgaard A.B.A., Møller-Sonnergaard J., Christrup L.L., Højsted J., Hansen R. (2001). Patients’ Evaluation of Shape, Size and Colour of Solid Dosage Forms. Pharm. World Sci..

[20] Walsh J., Ranmal S.R., Ernest T.B., Liu F. (2018). Patient Acceptability, Safety and Access: A Balancing Act for Selecting Age-Appropriate Oral Dosage Forms for Paediatric and Geriatric Populations. Int. J. Pharm..

[21] Ranmal S.R., O’Brien F., Lopez F., Ruiz F., Orlu M., Tuleu C., Walsh J., Liu F. (2018). Methodologies for Assessing the Acceptability of Oral Formulations among Children and Older Adults: A Systematic Review. Drug Discov. Today.

[22] Shariff Z.B., Dahmash D.T., Kirby D.J., Missaghi S., Rajabi-Siahboomi A., Maidment I.D. (2020). Does the Formulation of Oral Solid Dosage Forms Affect Acceptance and Adherence in Older Patients? A Mixed Methods Systematic Review. J. Am. Med. Dir. Assoc..

[23] Hofmanová J.K., Rajabi-Siahboomi A., Haque S., Mason J., Teckoe J., To D., Batchelor H.K. (2019). Developing Methodology to Evaluate the Oral Sensory Features of Pharmaceutical Tablet Coatings. Int. J. Pharm..

[24] Bogdahn M., Torner J., Krause J., Grimm M., Weitschies W. (2021). Influence of the Geometry of 3D Printed Solid Oral Dosage Forms on Their Swallowability. Eur. J. Pharm. Biopharm..

[25] Oshima T., Hori S., Maida C., Miyamoto E. (2006). Effect of Size and Shape of Tablets and Capsules on Ease of Grasping and Swallowing (1): Comparison between Elderly and Students. Iryo Yakugaku Jpn. J. Pharm. Health Care Sci..

[26] Yamamoto S., Taniguchi H., Hayashi H., Hori K., Tsujimura T., Nakamura Y., Sato H., Inoue M. (2014). How Do Tablet Properties Influence Swallowing Behaviours?. J. Pharm. Pharmacol..

[27] Schiele J.T., Penner H., Schneider H., Quinzler R., Reich G., Wezler N., Micol W., Oster P., Haefeli W.E. (2015). Swallowing Tablets and Capsules Increases the Risk of Penetration and Aspiration in Patients with Stroke-Induced Dysphagia. Dysphagia.

[28] Shulman K.I., Shedletsky R., Silver I.L. (1986). The Challenge of Time: Clock-drawing and Cognitive Function in the Elderly. Int. J. Geriat. Psychiatry.

[29] Folstein M.F., Folstein S.E., McHugh P.R. (1975). “Mini-Mental State” A Practical Method for Grading the Cognitive State of Patients for the Clinician. J. Psychiatry Res..

[30] Daniels S.K., Ballo L.A., Mahoney M.-C., Foundas A.L. (2000). Clinical Predictors of Dysphagia and Aspiration Risk: Outcome Measures in Acute Stroke Patients. Arch. Phys. Med. Rehabil..

[31] Daniels S.K., Brailey K., Priestly D.H., Herrington L.R., Weisberg L.A., Foundas A.L. (1998). Aspiration in Patients with Acute Stroke. Arch. Phys. Med. Rehabil..

[32] Benjamini Y., Hochberg Y. (1995). Controlling the False Discovery Rate: A Practical and Powerful Approach to Multiple Testing. J. R. Stat. Soc. Ser. B Methodol..

[33] Benjamini Y., Yekutieli D. (2005). False Discovery Rate–Adjusted Multiple Confidence Intervals for Selected Parameters. J. Am. Stat. Assoc..

[34] Swanlund S.L. (2010). Successful Cardiovascular Medication Management Processes as Perceived by Community-Dwelling Adults over Age 74. Appl. Nurs. Res..

[35] Charlesworth C.J., Smit E., Lee D.S.H., Alramadhan F., Odden M.C. (2015). Polypharmacy Among Adults Aged 65 Years and Older in the United States: 1988–2010. J. Gerontol. Ser..

[36] Schöttker B., Saum K.-U., Muhlack D.C., Hoppe L.K., Holleczek B., Brenner H. (2017). Polypharmacy and Mortality: New Insights from a Large Cohort of Older Adults by Detection of Effect Modification by Multi-Morbidity and Comprehensive Correction of Confounding by Indication. Eur. J. Clin. Pharmacol..

[37] Gnjidic D., Hilmer S.N., Blyth F.M., Naganathan V., Cumming R.G., Handelsman D.J., McLachlan A.J., Abernethy D.R., Banks E., Couteur D.G.L. (2012). High-Risk Prescribing and Incidence of Frailty Among Older Community-Dwelling Men. Clin. Pharmacol. Ther..

[38] Barnett K., Mercer S.W., Norbury M., Watt G., Wyke S., Guthrie B. (2012). Epidemiology of Multimorbidity and Implications for Health Care, Research, and Medical Education: A Cross-Sectional Study. Lancet.

[39] Vallet T., Belissa E., Laribe-Caget S., Chevallier A., Chedhomme F.-X., Leglise P., Piccoli M., Michelon H., Bloch V., Meaume S. (2018). A Decision Support Tool Facilitating Medicine Design for Optimal Acceptability in The Older Population. Pharm. Res..

[40] Jain S., Rosenbaum P.R., Reiter J.G., Ramadan O.I., Hill A.S., Hashemi S., Brown R.T., Kelz R.R., Fleisher L.A., Silber J.H. (2022). Defining Multimorbidity in Older Patients Hospitalized with Medical Conditions. J. Gen. Intern. Med..

[41] Clavé P., Rofes L., Carrión S., Ortega O., Cabré M., Serra-Prat M., Arreola V. (2012). Pathophysiology, Relevance and Natural History of Oropharyngeal Dysphagia among Older People. Nestlé Nutr. Inst. Work Ser..

[42] Lau E.T.L., Steadman K.J., Mak M., Cichero J.A.Y., Nissen L.M. (2015). Prevalence of Swallowing Difficulties and Medication Modification in Customers of Community Pharmacists. J. Pharm. Pract. Res..

[43] Souza L.F., Nascimento W.V., Alves L.M.T., Silva A.C.V., Cassiani R.A., Alves D.C., Dantas R.O. (2019). Medication Swallowing Difficulties in People without Dysphagia. Rev. Cefac.

[44] FDA Size, Shape, and Other Physical Attributes of Generic Tablets and Capsules. https://www.fda.gov/files/drugs/published/Size--Shape--and-Other-Physical-Attributes-of-Generic-Tablets-and-Capsules.pdf?next=/answers/six-tips-to-avoid-getting-pill-stuck-in-your-throat/avoid-pill-getting-stuck-in-throat/.

[45] Perkins A.C., Wilson C.G., Blackshaw P.E., Vincent R.M., Dansereau R.J., Juhlin K.D., Bekker P.J., Spiller R.C. (1994). Impaired Oesophageal Transit of Capsule versus Tablet Formulations in the Elderly. Gut.

[46] Franko D.L., Shapiro J., Gagne A. (1997). Phagophobia: A Form of Psychogenic Dysphagia a New Entity. Ann. Otol. Rhinol. Laryngol..

[47] FDA Guidance for Industry—Food-Effect Bioavailability and Fed Bioequivalence Studies. https://www.fda.gov/files/drugs/published/Food-Effect-Bioavailability-and-Fed-Bioequivalence-Studies.pdf.

[48] Ema Guideline on the Investigation of Bioequivalence. https://www.ema.europa.eu/en/documents/scientific-guideline/guideline-investigation-bioequivalence-rev1_en.pdf.

[49] Gallo S.H., McClave S.A., Makk L.J.K., Looney S.W. (1996). Standardization of Clinical Criteria Required for Use of the 12.5 Millimeter Barium Tablet in Evaluating Esophageal Lumenal Patency. Gastrointest. Endosc..

[50] Smith C.H., Logemann J.A., Burghardt W.R., Zecker S.G., Rademaker A.W. (2006). Oral and Oropharyngeal Perceptions of Fluid Viscosity Across the Age Span. Dysphagia.

[51] Ekström J., Khosravani N., Castagnola M., Messana L., Ekberg O. (2017). Saliva and the Control of Its Secretion. Dysphagia, Diagnosis and Treatment.

[52] Xu F., Laguna L., Sarkar A. (2019). Aging-related Changes in Quantity and Quality of Saliva: Where Do We Stand in Our Understanding?. J. Texture Stud..

[53] Marvola M., Vahervuo K., Sothmann A., Marttila E., Rajaniemi M. (1982). Development of a Method for Study of the Tendency of Drug Products to Adhere to the Esophagus. J. Pharm. Sci..

[54] McCloskey A.P., Penson P.E., Tse Y., Abdelhafiz M.A., Ahmed S.N., Lim E.J. (2022). Identifying and Addressing Pill Aversion in Adults without Physiological-related Dysphagia: A Narrative Review. Br. J. Clin. Pharmacol..

