# Supplementary material for: Impact of Tablet Size and Shape on the Swallowability in Older Adults
Source: Pharmaceutics. 2023 Mar 23;15(4):1042. doi: 10.3390/pharmaceutics15041042 (PMC10145850; doi:10.3390/pharmaceutics15041042)
Supplement: Supplementary file 1 [file pharmaceutics-15-01042-s001.zip › pharmaceutics-2259095-supplementary.pdf]

Table S1. Questions with possible answers for the evaluation of tablets' deglutition. Question one to five was to be answered by participants, while question six to eleven needed to be answered by the study team.

	Questions	Answer Options
	**Participant-reported outcomes**	
1.1.	How was the tablet to swallow?	a) Well.<br>b) Moderately.<br>c) Not.
1.2.	If the tablet was 'not' or 'moderately' to swallow. What is the reason or what are the reasons? (multiple choice)	a) The tablet was too big.<br>b) The tablet shape was unpleasant to swallow or impaired the deglutition.<br>c) Attempting to swallow or swallowing the tablet caused pain.<br>d) The tablet surface felt rough.<br>e) Further comments. (Possibility for free answers.)
2.	Did the tablet cause a foreign body sensation?	a) Yes, during attempt to swallow.<br>b) Yes, during deglutition.<br>c) No.
3.	Did you bite on the tablet before you did swallow it?	a) Yes.<br>b) No.
4.	Could you imagine swallowing this tablet once a day for a time period of one week?	a) Yes.<br>b) Yes, if really necessary.<br>c) No.
5.	Could you imagine to swallow this tablet once a day for a time period of several months?	a) Yes.<br>b) Yes, if really necessary.<br>c) No.
	**Researcher-reported outcomes**	
6.	Time needed to swallow the tablet.	In seconds.
7.	Water volume needed to swallow the tablet.	In milliliter.
8.	Did the participant show any signs of aspiration during or after the deglutition?	a) Yes, clearing one's throat.<br>b) Yes, coughing.<br>c) No.
9.	Did the participant drink water after having taken the cup of water from the mouth for the first time?	a) Yes, once.<br>b) Yes, multiple times.<br>c) No.
10.	How many attempts were needed to swallow the tablet?	a) Deglutition was not successful.<br>b) Deglutition was successful after the … attempt.<br>c) It was not visible.
11.	How was the facial expression of the participant during the deglutition?	a) Positive.<br>b) Neutral.<br>c) Negative.

English translation of the original German questions and answers.

A) <u>Vom Studienteilnehmer auszufüllen:</u>

1. Wie war die Tablette zu schlucken?

	☐	nicht	
Die Tablette war...	☐	mäßig	...schluckbar.
	☐	gut	

Falls Sie «nicht» oder «mäßig» angegeben haben:

Was ist der Grund bzw. was sind Gründe hierfür?

(Mehrfachauswahl - Sie können maximal sechs Kreuze setzen.)

☐ Die Tablette war zu groß.

☐ Die Form der Tablette war unangenehm bzw. hinderlich zum Schlucken.

☐ Die Tablette klebte im Mund und/oder Rachen.

☐ Der Versuch des Schluckens bzw. das Schlucken der Tablette verursachte Schmerzen.

☐ Die Oberfläche der Tablette fühlte sich rau an.

☐ Weitere Anmerkungen:

………………………………………………………………………………………………………

………………………………………………………………………………………………………

2. Hat die Tablette ein Fremdkörpergefühl hervorgerufen?

Die Tablette hat...:

☐ kein		
☐ beim Versuch des Schluckens ein	...Fremdkörpergefühl hervorgerufen.	
☐ beim Schlucken ein		

Falls Sie die Tablette nicht geschluckt haben, können Sie die Beantwortung dieses Teils des Fragebogens jetzt beenden.

3. Haben Sie auf die Tablette gebissen, bevor Sie diese geschluckt haben?

☐ Ja

☐ Nein

4. Könnten Sie sich vorstellen, die Tablette einmal pro Tag über einen Zeitraum von einer Woche zu schlucken?

☐ Nein

☐ Ja

☐ Ja, aber nur wenn unbedingt notwendig.

5. Könnten Sie sich vorstellen, die Tablette einmal pro Tag über einen Zeitraum von mehreren Monaten zu schlucken?

☐ Nein

☐ Ja

☐ Ja, aber nur wenn unbedingt notwendig.

Fragebogen zur Schluckstudie v1.0

Figure S1. Original German questions to be answered by participants after the deglutition of each individual tablet.

Vom Studienteam auszufüllen:

1. Zeit, die zum Schlucken benötigt wurde (MM:SS): _ _ : _ _

2. Wassermenge, die zum Schlucken der Darreichungsform benötigt wurde: _ _ _ mL

3. Zeigt der Proband Zeichen einer Aspiration:
   - ☐ Nein
   - ☐ Ja, Räuspern
   - ☐ Ja, Husten

4. Wird von dem Probanden Wasser nachgetrunken?
   - ☐ Nein
   - ☐ Ja, einmalig
   - ☐ Ja, mehrmals

5. Schluckvorgang gelingt...
   - ☐ nicht.
   - ☐ beim ……. (Anzahl der Schluckversuche) Schluckversuch.
   - ☐ War nicht zu erkennen.

6. Gesichtsausdruck des Probanden während des Schluckvorgangs:
   - ☐ positiv
   - ☐ neutral
   - ☐ negativ

Weitere Anmerkungen und Beobachtungen:

____

____

____

Figure S2. Original German questions to be answered by the study team after the deglutition of each individual tablet.

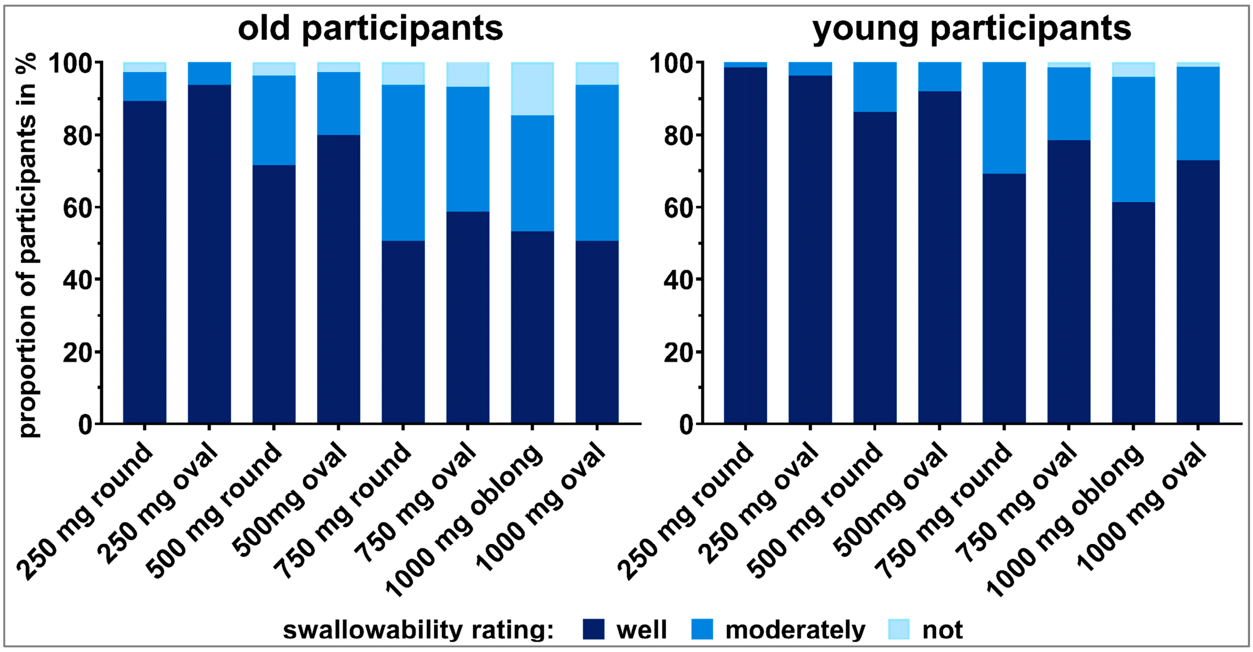

Figure S3. Raw data of swallowing ratings. Three evaluations for each study participant for each tablet are included. Data are shown separately for old (left panel) and young participants (right panel).

Table S2. Results of Fisher's exact test for comparisons of *well swallowable* vs. *not/moderately swallowable* data from old participants. Corrected *p*-values according to Benjamini Hochberg as well as corrected confidence intervals are given.

**Compared tablets (> indicating worse swallowability)**	***p*-value**	**odds ratio**	**lower 95% CI**	**upper 95% CI**	**corrected *p*-value**	**corrected lower CI: 98.5%**	**corrected upper CI: 98.5%**
250 mg: round < oval	0.4421	0.72	0.19	2.74	0.4421	0.14	3.78
500 mg: round < oval	0.1135	0.42	0.13	1.33	0.2270	0.10	1.75
750 mg: round < oval	0.3108	0.63	0.20	2.00	0.3501	0.15	2.63
1000 mg: oblong > oval	0.1407	2.25	0.70	7.22	0.2345	0.53	9.57
round: 250 mg < 500 mg	0.0638	2.94	0.90	9.65	0.1595	0.67	12.85
round: 250 mg < 750 mg	0.0009	7.52	2.19	25.85	0.0050*	1.62	34.82
oval: 250 mg < 500 mg	0.3151	1.71	0.46	6.32	0.3501	0.34	8.65
oval: 250 mg < 750 mg	0.0024	6.60	1.88	23.23	0.0080*	1.38	31.46
oval: 500 mg < 1000 mg	0.0010	7.35	2.16	25.03	0.0050*	1.60	33.64
oval: 750 mg < 1000 mg	0.2163	1.90	0.59	6.17	0.3090	0.44	8.19

Table S3. Results of Fisher's exact test for comparisons of *well swallowable* vs. *not/moderately swallowable* data from young participants. Corrected *p*-values according to Benjamini Hochberg as well as corrected confidence intervals are given. Even though the corrected *p*-values for the comparison between the round 250 mg tablets and the round 500 mg tablet as well as between the oval 500 mg and the oval 1000 mg tablets are <0.05, the comparisons cannot be seen as significant as the confidence intervals include an odds ratio of 1.00.

**Compared tablets (> indicating worse swallowability)**	***p*-value**	**odds ratio**	**lower 95% CI**	**upper 95% CI**	**corrected *p*-value**	**corrected lower CI: 98.0%**	**corrected upper CI: 98.0%**
250 mg: round > oval	0.5294	1.92	0.16	22.58	0.5294	0.11	35.80
500 mg: round < oval	0.3165	0.59	0.16	2.14	0.3956	0.13	2.72
750 mg: round < oval	0.1334	0.46	0.15	1.39	0.2668	0.12	1.71
1000 mg: oblong < oval	0.3780	0.72	0.24	2.16	0.4200	0.19	2.65
round: 250 mg < 500 mg	0.0164	10.11	1.16	88.00	0.0433	0.77	131.87
round: 250 mg < 750 mg	<.0001	34.91	4.10	297.49	0.001*	2.74	444.03
oval: 250 mg < 500 mg	0.1787	3.13	0.55	17.84	0.2978	0.40	24.71
oval: 250 mg < 750 mg	<.0001	8.33	1.60	43.29	0.0310*	1.18	58.90
oval: 500 mg < 1000 mg	0.0173	4.31	1.25	14.84	0.0433	0.99	18.70
oval: 750 mg < 1000 mg	0.2819	1.62	0.54	4.85	0.3956	0.44	5.96

Table S4. Results of Fisher's exact test for comparisons of *well swallowable* vs. *not/moderately swallowable* between age categories for the individual tablets. Corrected *p*-values according to Benjamini Hochberg are given.

**Compared tablets (old worse compared to young, exception oblong 1000 mg)**	***p*-value**	**odds ratio**	**lower 95% CI**	**upper 95% CI**	**corrected *p*-value**
250 mg round	0.0491	0.13	0.01	1.19	0.2640
500 mg round	0.1320	0.45	0.15	1.39	0.3362
750 mg round	0.2847	0.61	0.20	1.89	0.2640
250 mg oval	0.2101	0.35	0.06	2.00	0.3362
500 mg oval	0.3708	0.64	0.17	2.39	0.4238
750 mg oval	0.1289	0.44	0.14	1.38	0.2640
1000 mg oval	0.0791	0.38	0.12	1.18	0.2640
1000 mg oblong	0.5000	1.18	0.38	3.63	0.5000

Table S5. Results of Fisher's exact test for comparisons of *swallowable* vs. *not swallowable* data from old participants. Corrected *p*-values according to Benjamini Hochberg are given.

Compared tablets (> indicating worse swallowability)	*p*-value	odds ratio	lower 95% CI	upper 95% CI	corrected *p*-value
250 mg: round > oval	0.4808	-	-	-	0.6801
500 mg: round > oval	0.7239	0.92	0.12	7.08	0.7239
750 mg: round > oval	0.5447	1.28	0.26	6.36	0.6801
1000 mg: oval > oblong	0.3078	2.00	0.42	9.42	0.6801
round: 250 mg < 500 mg	0.5294	0.52	0.04	6.13	0.6801
round: 250 mg < 750 mg	0.1999	0.24	0.03	2.31	0.6801
oval: 250 mg < 500 mg	0.2262	0	-	-	0.6801
oval: 250 mg < 750 mg	0.1041	0	-	-	0.6801
oval: 500 mg < 1000 mg	0.5375	0.70	0.11	4.55	0.6801
oval: 750 mg < 1000 mg	0.7027	0.92	0.17	5.03	0.7239

Table S6. Results of Fisher's exact test for comparisons of *swallowable* vs. *not swallowable* data from young participants. Corrected *p*-values according to Benjamini Hochberg are given.

Compared tablets (> indicating worse swallowability)	*p*-value	odds ratio	lower 95% CI	upper 95% CI	corrected *p*-value
250 mg: round > oval	-	-	-	-	-
500 mg: round > oval	-	-	-	-	-
750 mg: round > oval	0.4808	0	-	-	0.7738
1000 mg: oval > oblong	0.7353	1.08	0.06	18.30	0.7738
round: 250 mg < 500 mg	-	-	-	-	-
round: 250 mg < 750 mg	-	-	-	-	-
oval: 250 mg < 500 mg	-	-	-	-	-
oval: 250 mg < 750 mg	0.4808	0	-	-	0.7738
oval: 500 mg < 1000 mg	0.5192	0	-	-	0.7738
oval: 750 mg < 1000 mg	0.7738	1.08	0.06	18.30	0.7738

Table S7. Results of Fisher's exact test for comparisons of *swallowable* vs. *not swallowable* between age categories for the individual tablets. Corrected *p*-values according to Benjamini Hochberg are given.

Compared tablets (old worse compared to young, exception oblong 1000 mg)	*p*-value	odds ratio	lower 95% CI	upper 95% CI	corrected *p*-value
250 mg round	0.5000	-	-	-	0.5000
500 mg round	0.2453	-	-	-	0.3315
750 mg round	0.0555	-	-	-	0.3315
250 mg oval	-	-	-	-	-
500 mg oval	0.2449	-	-	-	0.3315
750 mg oval	0.3046	3.27	0.31	33.84	0.3315
1000 mg oval	0.0947	3.25	0.32	33.41	0.3315
1000 mg oblong	0.3052	6	0.65	55.66	0.3315

Table S8. Water volume used to swallow the different tablets (up to max. 100 mL) by old participants and young participants.

	Old participants					Young participants				
	**Median [mL]**	**Mean [mL]**	**StdDev [mL]**	**Min [mL]**	**Max [mL]**	**Median [mL]**	**Mean [mL]**	**StdDev [mL]**	**Min [mL]**	**Max [mL]**
250 mg round	41.0	41.2	19.4	16.3	92.7	42.6	52.3	28.4	12.6	100.4
250 mg oval	29.3	34.5	16.7	13.0	77.7	35.5	37.2	22.5	0.0	99.5
500 mg round	35.7	37.4	19.0	13.0	89.0	40.4	41.9	21.8	8.7	99.4
500 mg oval	34.3	40.3	19.8	15.3	97.3	45.6	56.0	27.2	17.8	100.3
750 mg round	38.0	41.5	21.4	13.0	100.7	45.2	48.6	21.7	11.0	100.1
750 mg oval	37.0	44.6	20.8	14.0	87.7	53.3	58.6	24.4	16.8	100.1
1000 mg oblong	37.0	38.8	19.1	0.0	77.7	50.0	57.6	23.0	19.7	100.5
1000 mg oval	38.7	38.7	20.8	0.0	99.3	42.1	46.9	21.5	17.1	99.7

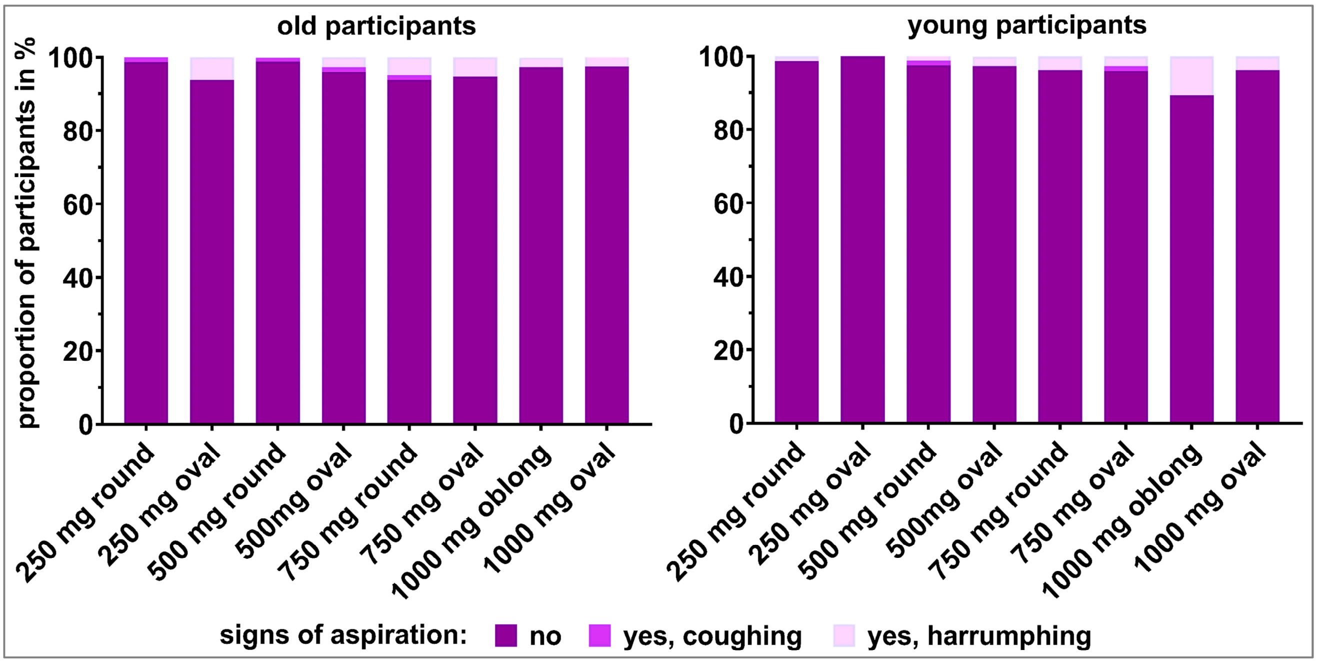

Figure S4. Signs of aspiration during and/or after the attempt or the actual deglutition of the individual tablets, separately for old (left panel) and young participants (right panel).

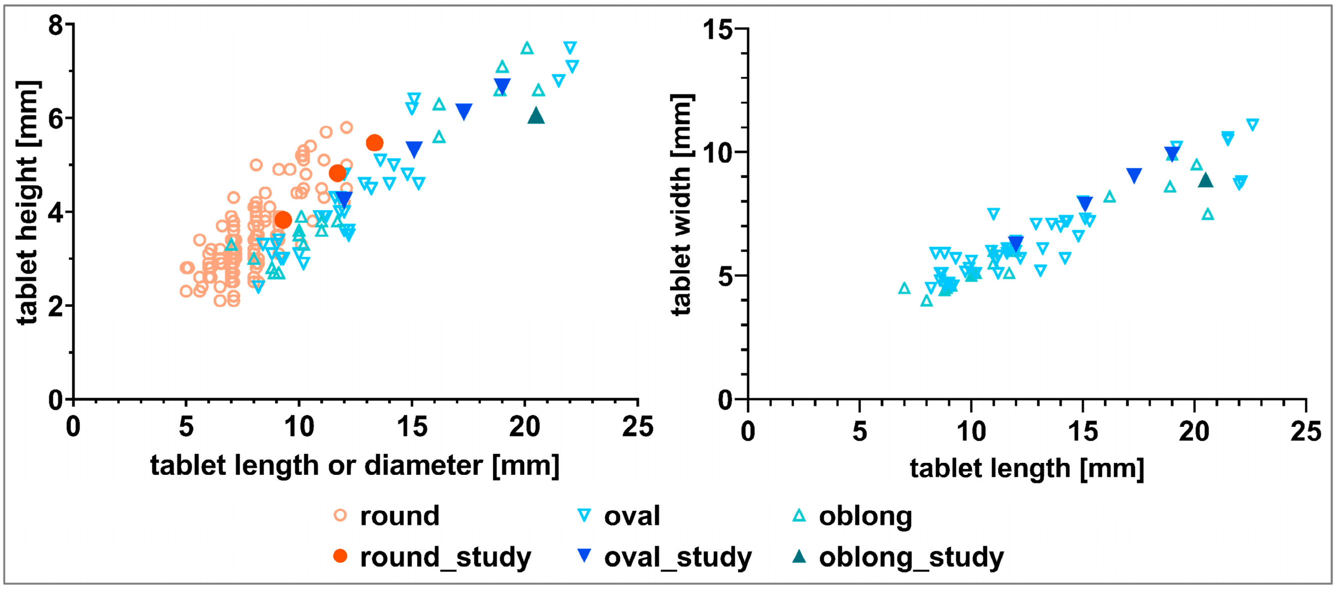

Figure S5. Tablet height (left panel) and tablet width (right panel) plotted against tablet length or tablet diameter, dependent on tablet shape being elongated (oval or oblong) or round, respectively. Data of tablets that are part of the 300 most prescribed drug products for older adults (65 years or older) in Germany are shown as symbol outlines. Filled symbols represent the dimensions of tablets being used in this study. Heights were missing for one round tablet, 18 oval tablets, and five oblong tablets.

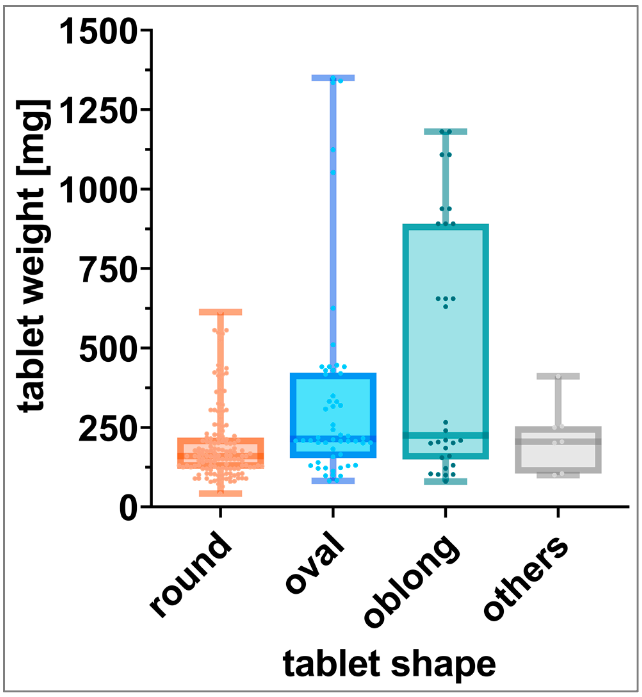

Figure S6. Tablet weight distribution by tablet shape for tablets that are part of the 300 most prescribed drug products for adults 65 years or older, in Germany. Round, oval, oblong, and other tablet shapes were represented by 61.0%, 23.3%, 12.6%, and 3.0%, respectively.
